# Supplementary material for: Cytoskeleton Protein BmACT1 Is Potential for the Autophagic Function and Nuclear Localization of BmAtg4b in Bombyx mori
Source: Cells. 2023 Mar 15;12(6):899. doi: 10.3390/cells12060899 (PMC10047584; doi:10.3390/cells12060899)
Supplement: Supplementary file 1 [file cells-12-00899-s001.zip › cells-2178314-supplementary.pdf]

Cytoskeleton protein BmACT1 is potential for the autophagic function and nuclear localization of BmAtg4b in *Bombyx mori*

Supplementary Figure S1

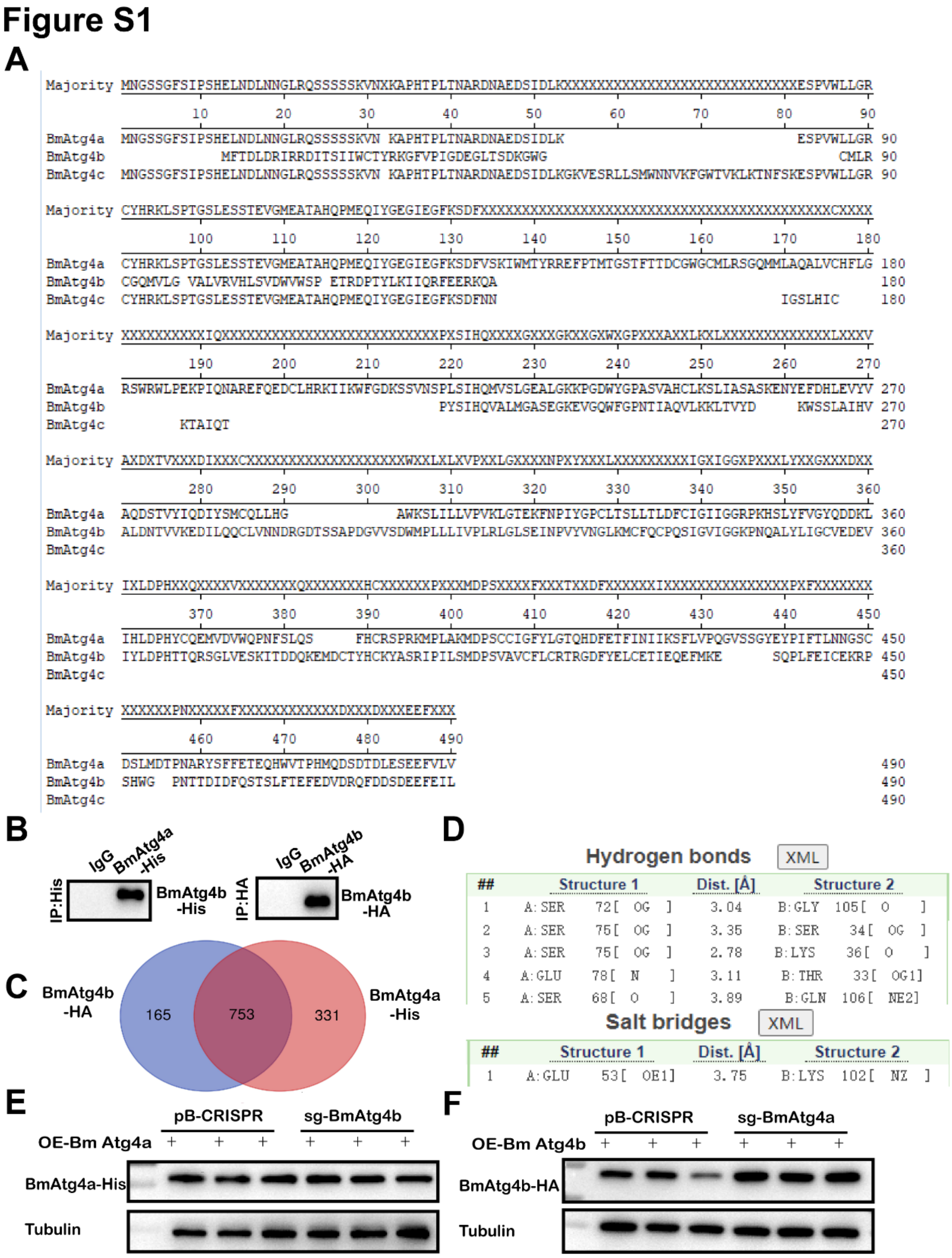

Figure S1. Different analysis of BmAtg4 protein:(A)Using MegAlign software, the amino acid sequences of three BmAtg4 proteins (BmAtg4a, BmAtg4b, and BmAtg4c)

of *B. mori* were compared and analyzed. (B) The overexpressed *BmAtg4a* and *BmAtg4b* were immunoprecipitated by the antibody of fused His or HA tag, respectively. (C) Immunoprecipitations of BmAtg4a and BmAtg4b were subjected to the analysis by mass spectrum. There were 753 proteins existed in common in the immunoprecipitate of BmAtg4a and BmAtg4b, while 165 proteins were specifically binding with BmAtg4a and 331 proteins were specifically binding with BmAtg4b. (D) As predicted by the https://www.ebi.ac.uk/msd-srv/prot_int/ website, BmAtg4a had a binding activity with BmAtg4b, and there were five hydrogen bonding sites and one salt bridge site between them. (E) WB of the overexpressed BmAtg4a-His protein in the *BmAtg4b* knockout BmN cells. (F) WB of the overexpressed BmAtg4b-HA protein in the *BmAtg4a* knockout BmN cells.

Supplementary Figure S2

Figure S2

A

Forward primer

```
ATCACGATTATTATCTATGTGGAACAATGTAAAATTTGGATGGACAGTAAAATTAAA
AACAAACTTTTCTAAAGAATCTCCGGTTTGGTTGTTGGGCCGATGTTATCACCGAAAA
TTAAGCCCAACTGGCTCACTGGAATCTTCCACAGAAGTGGGCATGGAAGCAACTGCA
CATCAACCAATGGAACAAATATATGGAGAGGGTATAGAAGGATTTAAATCAGATTTT
GTAAGTAAAATATGGATGACTTACAGAAGGGAATTCCCTACTATGACAGGTTCAACT
TTTACAACTGATTGTGGTTGGGGTTGTATGCTTCGAAGTGGACAAATGATGTTGGCTC
AGGCTTTAGTTTGCCATTTCCTAGGCCGTTCATGGAGATGGTTACCTGAGAAACCCAT
TCAAAATGCTAGAGAATTTCAAGAAGATTGTTTACATAGAAAAATTATAAAGTGGTTT
GGAGATAAGTCTTCAGTTAATAGTCCTCTGTCAATACATCAGATGGTGAGTTTGGGAG
AAGCTTTAGGCAAGAAGCCAGGTGATTGGTATGGCCCTGCATCTGTAGCACATTGTTT
GAAATCTCTAATTGCATCAGCTTCAAAAGAGA
```

Revers primer

```
(Control) ACTGATTGTGGTTGGGGTTGTATGCTTCGAAGTGGACAAATGATGTTGG
(1)       ACTGATTGTGGTTGGG-------------AAGTGGACAAATGATGTTGG
(2)       ACTGATTGTGGTTGGGGTTGTATGCTT--AAGTGGACAAATGATGTTGG
(3)       ACTGATTGTGGTTGGGGTTGTATGC----AAGTGGACAAATGATGTTGG
(4)       ACTGATTGTGGTTGGGGTTGTA-------AAGTGGACAAATGATGTTGG
(5)       ACTGATTGTGGTTGGGGT-----------AAGTGGACAAATGATGTTGG
(6)       ACTGATTGTGGTTGGGGTTGTATGCTTC-AAGTGGACAAATGATGTTGG
(7)       ACTGATTGTGGTTGGG---------------GTGGACAAATGATGTTGG
(8)       ACTGATTGTGGTTGGGG--------------GTGGACAAATGATGTTGG
(9)       ACTGATTGTGGTTGGGGTTGTATGCTTCG-AGTGGACAAATGATGTTGG
(10)      A--------------------------------------ATGATGTTGG
```

B

Forward primer

```
CTTTTTTCTTGGACATCCTCGTCAGCAACATTGCACTCCCTTTTGTCCTTCAATACTACA
TTTTTCCACATTTAATTAGATTAAATAAAATTAAACAGAAAATAAATGTTCACAGATTTG
GATCGTATTAGAAGAGATATCACATCAATAATATGGTGTACGTACAGAAAAGGCTTTGTA
CCAATTGGTGATGAAGGTTTGACTTCTGATAAAGGATGGGGCTGTATGTTACGATGTGG
ACAGATGGTATTAGGTGTTGCTCTTGTCAGAGTACACTTATCTGTTGACTGGGTATGGTC
ACCAGAAACAAGGTAATTGTATTTAAATGATTGCAAAATAGTTAATTTTCATAATACTTC
TTTATCAATTAGTATCTTGTTGAATTCTTGCAATTTCTTTTAATAAATGGAGTTAATTTTTC
TAATGTTGTGTACCTTTATGTAGCTAATGGTGAAAGGTCTCTATTTCACTTTATATTATTAT
TGAATAAATTATAAAAAATGTGAAGATATAAAATTTTACAGGGATCCAACTTATTTAAAG
ATCATACAAAGGTTTGAAGAAAGGAAACAAGCACCTTATTCTATCCA
```

Revers primer

```
(Control) CCAATTGGTGATGAAGGTTTGACTTCTGATAAAGGATGGGGCTGTATGTT
(1)       CCAATTGGTGATGAAGGTTTGACT-------------GGGGCTGTATGTT
(2)       CCAATTGGTGATGAAGGTTTGACTTCCG---AAGGATGGGGCTGTATGTT
(3)       CCAATTGGTGATGAAGGTTTGACTTCTGACAA-GGATGGGGCTGTATGTT
(4)       CCAATTGGTGATGAAGGTTTGACTTCT------GGATGGGGCTGTATGTT
(5)       CCAATTGGTGATGAAGGTTTGACTTCTG----------GGGCTGTATGTT
(6)       CCAATTGGTGATGAAGGTTTGACTTCT-----AGGATGGGGCTGTATGTT
(7)       CCAATTGGTGATGAAGGTTTGACTTCTGATA--GGATGGGGCTGTATGTT
(8)       CCAATTGGTGATGAAGGTTTGACTTCTGATAC------------TATGTT
(9)       CCAATTGGTGATGAAGGTTTGACTTCTGAT----GATGGGGCTGTATGTT
(10)      CCAATTGGTGATGAAGGTTTGACTTCTGATAAA-----------------
```

Figure S2. Knockout efficiencies of *BmAtg4a* and *BmAtg4b*. (A) Partial genome sequence of *BmAtg4a* containing the knockout sites and the knockout efficiency. (B) Partial genome sequence of *BmAtg4b* containing the knockout sites and the knockout efficiency. Note: Primers are indicated in the green section. Nucleotides in yellow background are the sequence of sgRNA; Protospacer adjacent motifs (PAMs) are red with yellow background.

Supplementary Figure S3

Figure S3

A

Forward primer

```
AGTTCTTTAGACAGGTAAGTTAGTGTAATTGTGTACATTTCGCGTGTATTATCGAGCGCG
ATCGTGGAGAGGCGGGGGCGACCCGGCTCGCCTTGTATGGAGCGCGGCCGCGGCTCA
CGCGCGTCCTATATAAGAAACCGGCGCCGGGAACCGCCGCACTCGCAGTCTCGCTGCC
GTAGAGTCAATACCAGCGTCCCCAACACAAACACACACAAAGTTCAAGATGTGCGAC
GACGATGTTCGTGCGCTTGTAGTCGACAATGGCTCCGGCATGTGCAAGGCCGGTTTCG
CCGGGGACGACGCGCCCCGGGCCGTCTTCCCGTCCATCGTGGGTCGCCCTCGCCACCA
GGGGCTGATGGTCGGTATGGGCCAGAAAGACTCCTACGTGGGTGACGAGGCCCAGAG
CAAGAGAGGTATCCTCACTCTGAAGTACCCCATCGAGCACGGTATCATCACCAACTGG
GATGACATGGAGAAGATCTGGCACCACACCTTCTACAATGAGCTGCGTGTC
```

Revers primer

```
(Control) CGTGCGCTTGTAGTCGACAATGGCTCCGGCATGTGCAAGGCCGGTTTCGCCGGG
      (1) CGTGCGCTTGTAGTCGACAATGGCTCCGGC--------GGCCGGTTTCGCCGGG
      (2) CGTGCGCTTGTAGTCGACAATGGCTC-----TGTGCAAGGCCGGTTTCGCCGGG
      (3) CGTGCGCTTGTAGTCGACAATGGCTCCGGCATGTGCAAGGCCGGTTTCGCCGGG
      (4) CGTGCGCTTGTAGTCGACAATGGCTCCGGCGTG----------CGGTTTCGCCGGG
      (5) CGTGCGCTTGTAGTCGACAATGGCTCCGGC--------GGCCGGTTTCGCCGGG
      (6) CGTGCGCTTGTAGTCGACAATGGCTCCGGC-----------CGGTTTCGCCGGG
      (7) ---GCGCTTGTAGTCGACAATGGCTCCGGC--------GGCCGGTTTCGCCGGG
      (8) CGTGCGCTTGTAGTCGACAATGGCTCCGGC--------GGCCGGTTTCGCCGGG
      (9) CGTGCGCTTGTAGTCGACAATGGCTCCGGCATGTGCA-----------------
     (10) CGTGCGCTTGTAGTCGACAATGGC--------------------------------
```

B

Forward primer

```
TGGTAAAGGTGAGTGCGTGTCGCGGCGGGGCGGGGCGGTGCGTGGCGGGGCGGGGC
GGGGCGGGGCGAGGGTGCCGGCTCTATATAGCGCGCCGGACGCCGTGCGCGACCACT
GCTGCCCGCCCGCTCTCTGCCGCACGCACGCCGCCCGGTACAACCACCCACACCAGC
CCTACACCAGTTGAGAAGTAACAGCGTGACCGAGATCAGCAGCCATGTGTGACGATGA
TGCGGGCGCGCTCGTCGTCGACAACGGATCCGGCATGTGCAAGGCTGGATTCGCGGG
GGACGACGCCCCTCGAGCCGTGTTCCCTTCCATAGTGGGCCGCCCGCGCCACCAGGGC
GTCATGGTCGGCATGGGCCAAAAAGATTCGTATGTAGGTGACGAAGCCCAGAGCAAG
AGAGGTATCCTCACTCTAAAGTACCCCATCGAGCACGGGATAATCACCAACTGGGATG
ACATGGAGAAGATCTGGCATCACACGTTCTACAATGAGCTGCGCGTCGCTCCCGAGGA
GCACCCAATCCTGCTGACCGAGGCACCCCTGAACCCGAAGGCCAACAGAGAGAAGAT
GACTCAGATCATGTTCGAGACGTT
```

Revers primer

```
(Control) GTGACGATGATGCGGGCGCGCTCGTCGTCGACAACGGATCCGGCATGTGCAAG
      (1) GTGACGATGATGCGGGCGCGCTCGTCGTCG-CAACGGATCCGGCATGTGCAAG
      (2) GTGACGATGATGCGGGCGCGCTCGTCGTCG-CAACGGATCCGGCATGTGCAAG
      (3) GTGACGATGATGCGGGCGCGCTCGTCGTCG-CAACGGATCCGGCATGTGCAAG
      (4) GTGACGATGATGCGGGCGCGC----------CAACGGATCCGGCATGTGCAAG
      (5) GTGACGATGATGCGGGCGCGCTCGTC------CAACGGATCCGGCATGTGCAAG
      (6) GTGACGATGATGCGGGCGCGCTCGTCGTC--CAACGGATCCGGCATGTGCAAG
      (7) GTGACGATGATGCGGGCGCGCTCGTCG---ACAACGGATCCGGCATGTGCAAG
      (8) GTGACGATGATGCGGGCGCGCTCGTCGTC---CAACGGATCCGGCATGTGCAAG
      (9) GTGACGATGATGCGG-----------------------------GCATGTGCAAG
     (10) GTGACGA-----------------------------GGATCCGGCATGTGCAAG
```

Figure S3. Knockout efficiencies of *BmACT1* and *BmACT2*. (A) Partial genome sequence of *BmACT1* containing the knockout sites and the knockout efficiency. (B) Partial genome sequence of *BmACT2* containing the knockout sites and the knockout efficiency. Note: Primers are indicated in the green section. Nucleotides in yellow background are the sequence of sgRNA; PAMs are red with yellow background.

Supplementary Table S1 Primer sequences of sgRNAs

Genes	Forward 5’-3’	Reverse5’-3’
BmAtg4a	AAGTTGGGGTTGTATGCTTCGAAG	AAACCTTCGAAGCATACAACCCCA
BmACT2	AAGTGGCGCGCTCGTCGTCGACAA	AAACTTGTCGACGACGAGCGCGCC
BmAtg4b	AAGTGTTTGACTTCTGATAAAGGA	AAACTCCTTTATCAGAAGTCAAAC
BmACT1	AAGTGGCTCCGGCATGTGCAAGGC	AAACGCCTTGCACATGCCGGAGCC

Note: AAGT and AAAC were restricted endonuclease sites

Supplementary Table S2 Primer sequences for qPCR

Genes	Forward 5’-3’	Reverse 5’-3’
ie1	CGTGAGCAATGTGGTGTACG	AATTTTGTGCAGCCGTCTCG
gp64	ACACGTGCAACAAATCGTGG	ATACTCACGCCGTCTCGATG
helicase	ACAGCGGAGAGTCCAACAAG	TACGACTCGCTGCACTGTTT
BmGAPDH	CGTGAGCAATGTGGTGTACG	GCTGCCTCCTTGACCTTTTGC
BmAtg4a	GGCTTTAGTTTGCCATTTCCT	CCATACCAATCACCTGGCTTC
BmAtg4b	TAGATCCCCATACAACTCAACG	TGGCTTTCTTTCATAAACTCCT
Bmrp49	CAGGCGGTTCAAGGGTCAATAC	TGCTGGGCTCTTTCCACGA

Supplementary Table S3 and Table S4 are attached in the Supplementary File.
